# Single-Cell Transcriptional Profiling Reveals Sex and Age Diversity of Gene Expression in Mouse Endothelial Cells

**DOI:** 10.3389/fgene.2021.590377

**Published:** 2021-02-17

**Authors:** Xianxi Huang, Wenjun Shen, Stefan Veizades, Grace Liang, Nazish Sayed, Patricia K. Nguyen

**Affiliations:** ^1^Department of Critical Care Medicine, The First Affiliated Hospital of Shantou University Medical College, Shantou, China; ^2^Division of Cardiovascular Medicine, Stanford University, Stanford, CA, United States; ^3^Stanford Cardiovascular Institute, Stanford, CA, United States; ^4^Department of Bioinformatics, Shantou University Medical College, Shantou, China; ^5^Center for Biomedical Informatics Research, Stanford University, Stanford, CA, United States; ^6^Edinburgh Medical School, College of Medicine and Veterinary Medicine, University of Edinburgh, Edinburgh, United Kingdom; ^7^Cardiology Section, Department of Veteran Affairs, Palo Alto, CA, United States

**Keywords:** single-cell sequencing, endothelial cells, sex, age, cardiovascular disease

## Abstract

Although it is well-known that sex and age are important factors regulating endothelial cell (EC) function, the impact of sex and age on the gene expression of ECs has not been systematically analyzed at the single cell level. In this study, we performed an integrated characterization of the EC transcriptome of five major organs (e.g., fat, heart-aorta, lung, limb muscle, and kidney) isolated from male and female C57BL/6 mice at 3 and 18 months of age. A total of 590 and 252 differentially expressed genes (DEGS) were identified between females and males in the 3- and 18-month subgroups, respectively. Within the younger and older group, there were 177 vs. 178 DEGS in fat, 305 vs. 469 DEGS in heart/aorta, 22 vs. 37 DEGS in kidney, 26 vs. 439 DEGS in limb muscle, and 880 vs. 274 DEGS in lung. Interestingly, LARS2, a mitochondrial leucyl tRNA synthase, involved in the translation of mitochondrially encoded genes was differentially expressed in all organs in males compared to females in the 3-month group while S100a8 and S100a9, which are calcium binding proteins that are increased in inflammatory and autoimmune states, were upregulated in all organs in males at 18 months. Importantly, findings from RNAseq were confirmed by qPCR and Western blot. Gene enrichment analysis found genes enriched in protein targeting, catabolism, mitochondrial electron transport, IL 1- and IL 2- signaling, and Wnt signaling in males vs. angiogenesis and chemotaxis in females at 3 months. In contrast, ECs from males and females at 18-months had up-regulation in similar pathways involved in inflammation and apoptosis. Taken together, our findings suggest that gene expression is largely similar between males and females in both age groups. Compared to younger mice, however, older mice have increased expression of genes involved in inflammation in endothelial cells, which may contribute to the development of chronic, non-communicable diseases like atherosclerosis, hypertension, and Alzheimer's disease with age.

## Introduction

The endothelium comprises a single monolayer of cells that lines the cardiovascular and lymphatic system, serving as the interface between tissue walls and the blood and lymph, respectively. Although once considered a passive conduit for nutrient and waste exchange, endothelial cells (ECs) are now recognized as active regulators of coagulation, inflammation, vascular tone, metabolism, and tissue repair. Endothelial cells, however, are not identical in their structure and function across organ systems. An organ's phenotype as well as its microenvironment play an important role in shaping vascular development during embryogenesis as well as vascular repair after injury, which in turn alters the morphology and behavior of ECs within individual organs and across various organs. Interestingly, EC heterogeneity may be maintained even after removal from their microenvironment as shown in previous studies showing that ECs from different organs respond uniquely to pro-inflammatory cytokines, such as tumor necrosis factor alpha, interleukin-1 beta and the bacterial product lipopolysaccharide, when administered *in vitro* (Booth et al., 2004; Gutierrez et al., 2013). Whether age and sex add additional layers of heterogeneity has not been systematically evaluated.

As the gatekeepers of vascular health, it is not surprising that injury to endothelial cells in the arteries, capillaries and veins has been associated with a myriad of diseases affecting the brain [e.g., multiple sclerosis (Barak et al., 2017), stroke (Budhiraja et al., 2004)], cardiovascular system [e.g., coronary artery disease (Johnson and Nangaku, 2016), vasculitis (Perticone et al., 2010)], lung [e.g., asthma, COPD (Dabiré et al., 2012), pulmonary hypertension (Timmerman and Volpi, 2013)], kidney [e.g., diabetic kidney disease (Molema, 2010), hypertensive kidney disease (Muller et al., 2002)], and muscle [e.g., Duchenne's muscular dystrophy (Derada Troletti et al., 2019), age-associated sarcopenia (Cereda et al., 2013)]. Importantly, many of these diseases have age (e.g., old vs. young) and sex dimorphisms in their prevalence, manifestation, and outcome. The biological reasons underlying these clinical observations remain poorly understood.

We hypothesize that the phenotypic similarities and differences in EC structure and function across various organs are reflected in their global gene expression and show a pattern of age and sexual dimorphism. While we are not the first group to evaluate the EC global gene expression across organs (Feng et al., 2019), we provide an unbiased, systematic, and comprehensive comparison of EC transcriptomics based on sex and age within the tissue microenvironment of 5 major organs (e.g., fat, heart and aorta, lung, limb muscle, and kidney) harvested from the same mice, using state-of-the-art single cell technology. We identify shared and organ-specific gene signatures for ECs in males and females across different age groups. Findings from this study will not only provide a reference guide for the gene expression of ECs across multiple organs in males and females, but may also provide valuable insight into the potential mechanisms that underlie why the patterns of certain diseases may vary by sex and age and may facilitate the development of personalized approach to diagnosis and treatment.

## Materials and Methods

### Data Source and Identification of Differentially Expressed Genes

Single cell transcript data was obtained from the database generated by the Tabula Muris Consortium et al. (2018) and Tabula Muris Consortium (2020) (https://figshare.com/projects/Tabula_Muris_Transcriptomic_characterization_of_20_organs_and_tissues_from_Mus_musculus_at_single_cell_resolution/27733).

The data was processed using Seurat V2. The expression of Cdh5 and Pecam1 were used to identify the endothelial cells in each tissue in males and females in the young and old cohort. Of the 20 organs, only the following five organs contained sufficient cell numbers for analysis: (1) fat, (2) heart and aorta, (3) lung, (4) limb muscle, and (5) kidney. Only cells that expressed both transcripts in these five organs were merged into a Seurat object and analyzed for differential expression at a cut off of log2 fold change >1 and adjusted *p* < 0.05 (Benjamin Hochberg).

### GO and Pathway Enrichment Analysis

Gene ontology and pathway enrichment analysis was performed using EnrichR (https://amp.pharm.mssm.edu/Enrichr3; Chen et al., 2013; Kuleshov et al., 2016), *ClusterProfiler*, and the Gene Ontology Consortium website (http://geneontology.org/). Specifically, all differentially expressed genes for males and females from all organs were analyzed to generate Figures 4–9. The *p*-value is computed using the Fisher exact test and represents the probability of having at least × genes out of y total genes in the list annotated by the GO term, given the proportion of genes in the whole genome annotated by the GO term. For Figure 4 for the analysis of all ECs, we used Enrichr, which uses a combined score that is computed by multiplying the unadjusted *P*-value with the *Z*-score that is calculated by assessing the deviation from the expected rank. For Figures 5–9 for the analysis of ECs in each organ, we used Cluster Profiler, which computes a gene ratio that represents the number of genes in our input list associated with a given GO term divided by the total number of input genes. All graphs were plotted in Prism 7, and a heatmap was plotted in R 3.6.1 (https://www.r-project.org).

### Aortic Endothelial Cell Isolation, qPCR, and Western Blot

To verify findings from single cell transcriptomics, we performed qPCR on aortic ECs isolated from young and old C57Bl/6J mice. To obtain ECs for *in vitro* culture, we extracted aortas from mice (*n* = 6 per group) and digested them using Liberase (e.g., 5 mg dissolved in 10 ml DMEM/F12 medium to achieve a concentration of 1 mg/ml). We collected the cell pellet and resuspended it in EGM medium with 5%FBS and Pen strep. The resuspended cells were then placed in 24 well-plates coated with gelatin. Media was changed daily. After 1 week, wells containing confluent cells were trypsinized and re-plated into six well-plates. After another week of expansion, cells were trypsinized and collected for RNA extraction, cDNA synthesis, and qPCR using standard protocols. We used the following primers: (1) Lars2, (2) S100a8, (3) S100a9, and (4) genes belonging to the WNT pathway (e.g., FZD4, PFN1, PSMA2, PSMA7, PSMB8, and PSMB).

In addition to PCR, using standard protocols, we performed Western blot on aortic ECs to determine the protein expression of selected genes. Briefly, endothelial cells were harvested and lysed for Western blot analysis. Protein was loaded onto 4–15% Tris gels (Bio-red) at 100 V for 60 min. The separated proteins were transferred onto a polyvinylidene fluoride (PVDF) membrane (Bio-red, 0.2 μm). The PVDF membrane was then blocked with 5% skim milk powder at room temperature for 2 h, washed with PBS for 3 min, and incubated overnight at room temperature with the following rabbit anti-mouse antibodies: (1) anti-Lars2 (Proteintech, 1:500, 17170-1-AP), (2) anti-Profilin-1 (Invitrogen, 1: 1,000, 11680-1-AP), (3) anti-Frizzled4 (Invitrogen, 1: 300, PA5-41972), (4) anti-S100a8 (Invitrogen, 1:100PA5-79948), (5) anti-S100a9 (Invitrogen, 1:200, 14226-1-AP), and (6) anti-GAPDH (Invitrogen, 1:2,000, # 39-8600). The membrane was then incubated with anti-rabbit secondary antibody (Jackson immunoresearch, 1:5,000, 111-035-144) for 2 h in room temperature and washed by TBST three times. Protein expression was detected using enhanced chemiluminescence. The relative expression of the target protein was defined as the ratio of average OD value of target protein bands to that of the internal reference GAPDH.

### Statistical Analysis

All statistical analysis was performed by GraphPad Prism software (version 7) and R software (version 3.6.1). All *p*-values were adjusted for multiple comparisons. Adjusted *p* < 0.05 was considered statistically significant.

## Results

### Data Source and Analysis

**Figure 1:**
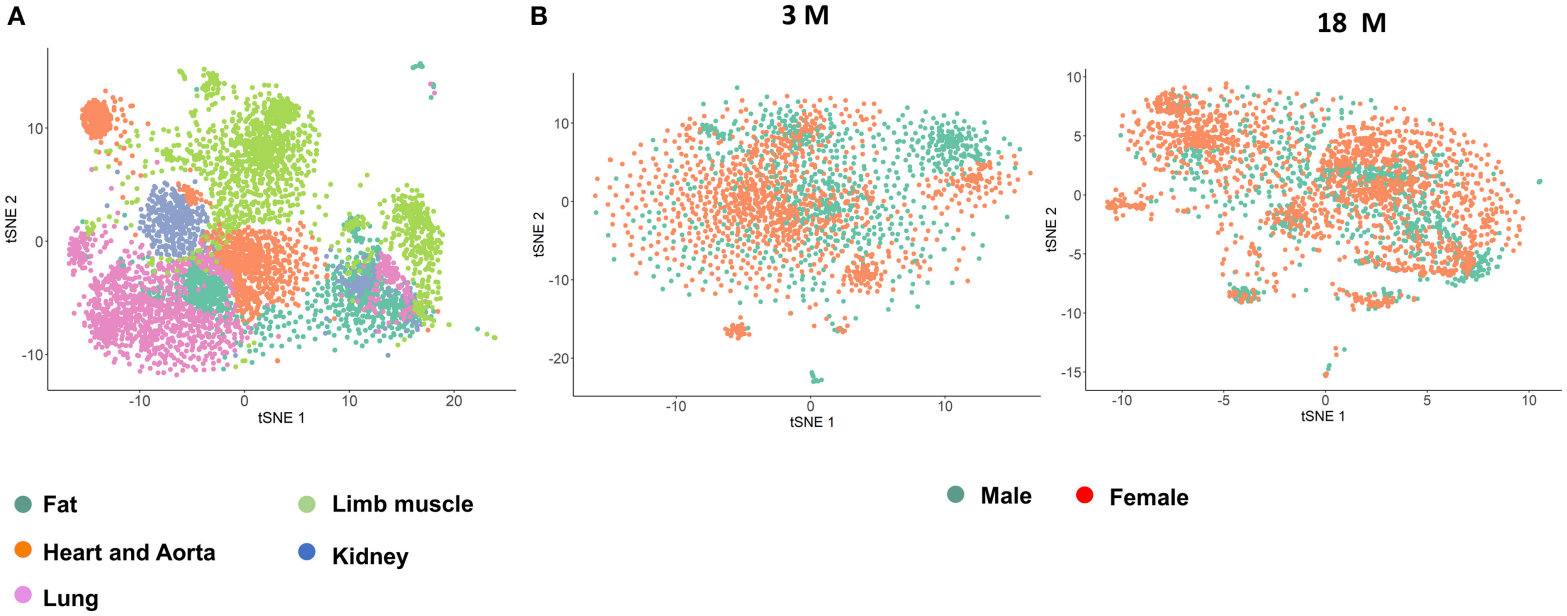
*T-SNE* visualization of endothelial cells (ECs) from single cell sequencing. **(A)** *t***-**SNE plot of all ECs collected by single cell transcriptomics and colored by organ; **(B)** *t***-**SNE plot of all ECs colored by sex in the 3 month (*left*) and 18 month (*right*) age groups.

Original data from the Tabula Muris Consortium (e.g., *Tabula Muris* and *Aging Transcriptomic Atlas*) was obtained (Tabula Muris Consortium et al., 2018; Tabula Muris Consortium, 2020). Information on the following 5 organs were analyzed from 4 male and 3 female mice in the 3 month group, and 2 males and 4 females in the 18 month group: fat, heart and aorta, lung, limb muscle, and kidney (Supplementary Figure 1A). To identify distinct cell populations based on shared and unique patterns of gene expression, we performed dimensionality reduction and unsupervised cell clustering methods. EC lineage genes, Pecam1 and Cdh5, were used as markers to identify the ECs (Figure 1; Tabula Muris Consortium, 2020). Cell counts for each organ stratified by age and sex are shown in Supplementary Figure 1B. Profiles of 4,883 cells analyzed in the Seurat V2 by unsupervised analysis revealed that most cells are grouped by their parent organs. There was a sub-cluster of ECs from the heart and aorta as well as another sub-cluster composed of all organs except the heart and aorta that diverged from the primary cluster. Analysis by sex in each age group, did not reveal distinct clusters, suggesting that the majority of the transcriptome in male and female ECs is similar across age groups.

### Differentially Expressed Genes in All ECs Based on Age and Sex

**Figure 2:**
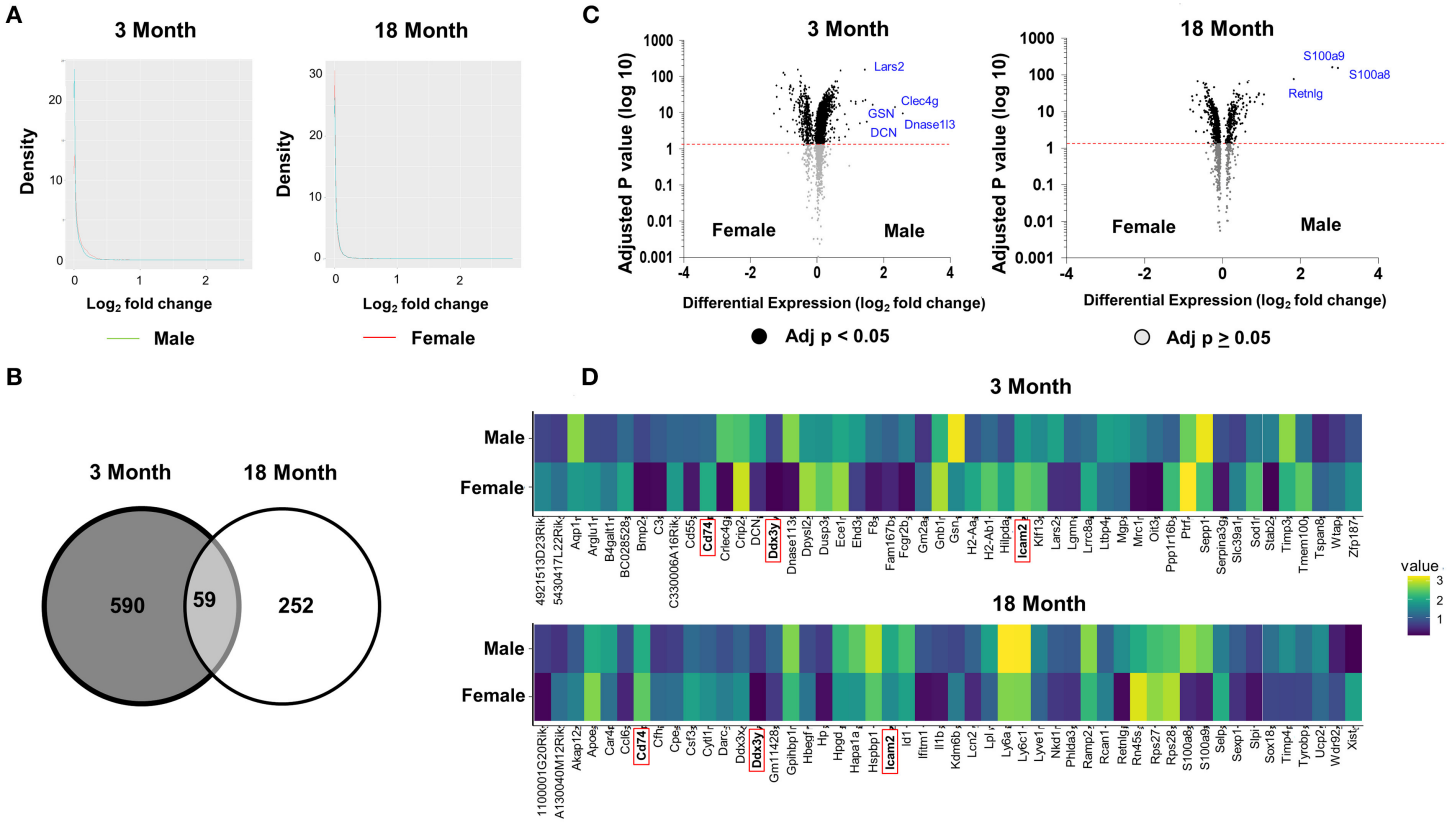
Differential analysis reveals age and sex dimorphism in ECs. **(A)** A comparison of density plots of differential gene expression reveals the expression of the majority of genes was not significantly different in ECs isolated from males and females for both age groups (log fold change <1). Genes up-regulated in male mice plotted in aqua. Genes up-regulated in female mice plotted in red. **(B)** Venn diagram showing 590 and 252 genes that are differentially expressed (adjusted *p* < 0.05) between females and males in the 3 and 18 month group, respectively. Of those genes that are differentially expressed, there are 59 shared genes that are differentially expressed between the sexes in both age groups. **(C)** The most statistically upregulated genes with the largest fold-change in males at 3 months were: Lars2, GSN, Clec4g, DCN, and Dnase1l3 at 3 months. The most statistically upregulated genes with the largest fold-change in males at 18 months was S100a9, S100a8, and Retnlg. The most statistically significant genes with the larges fold change include Xist, Ddx3x, and Cfh, which are upregulated in females, and S1009, S1008, and Retinlg, which are upregulated in males at 3 months *(left)*. The most statistically significant genes with the larges fold change include H2Ab1, Ppp1r16b, and Tmem100B in females and Dnase1l3, Clec4g, Lars2, GSN, and DCN in males at 18 months *(right)*. **(D)** Heat map showing the expression levels of 50 genes with the largest fold difference between males and females at 3 months (*top)* and 18 months (*bottom*). Of the 50 top genes, the following three genes have the largest fold difference and shared between the young and old group (*highlighted in red*): Cd74, DDx3y, and Icam2.

Subsequent analyses focused on comparing the patterns of differential gene expression between male and female in young and old age group. Consistent with tSNE visualization analysis of all ECs by age and sex, density plots showed that the majority of genes have <50% difference in expression between males and females (log_2_ fold <1) (Figure 2A). A total of 590 and 252 differentially expressed genes (DEGS) were identified between females and males in the 3- and 18-month subgroups, respectively, with 59 shared genes between young and older mice that were sexually dimorphic (Figure 2B and Supplementary Table 1). These genes are involved in angiogenesis (e.g., Acvrl1, Lrg1, Ptprb, and Tmem100), immunity and inflammation (e.g., Adamts1, Cd74, Cebpb, Ctla2a, DCN, Fcgrt, H2-Ab1, Icam2, Kdm6b, Lcn2, Nfkbia, Nfkbiz, and Sgk1), cellular chemotaxis (e.g., Ecscr, Gpr56, Pcdh1, and Tmsb10), endothelial specific function (e.g., Apold1), smooth muscle cell differentiation (e.g., Crip2), and cellular growth and development (e.g., Bmpr2, Ccdc85b, Egr1, Fosb. Id3, Oaz1, Pfkbfb3, and Tspan8), apoptosis (e.g., Gas5 and Phlda3), and lipid metabolism (e.g., Thrsp). Of note, genes with the largest fold change (>1.4-fold) included Dnase1l3, Clec4g, Lars2, GSN, and DCN, which were significantly upregulated in males at 3 months. Of these, Clec4g and GSN are important in the immune response, DCN is related to angiogenesis, Lars2 is involved in mitochondrial function, and DNASE1l3 regulates apoptosis (Figure 2C and Supplementary Table 2). In contrast, three genes including RETNLG, S100A8, and S100A9, which are involved in the regulation of immune function and inflammation, were significantly upregulated in males >18 months. When comparing the gene expression profiles across the age groups, we find the following two genes with shared sexual dimorphism across age: (1) Cd74, a cell surface receptor for cytokine macrophage migration inhibitory factor that is involved in apoptosis, immune response and cell migration (Fan et al., 2011; Le Hiress et al., 2015; Gil-Yarom et al., 2017); and (2) ICAM2, intracellular adhesion molecule 2, that mediates adhesive interactions important for immune response and surveillance and angiogenesis (Figure 2D; Huang et al., 2005; Halai et al., 2014).

### Differentially Expressed Genes of ECs in the Five Major Organs Based on Age and Sex

**Figure 3:**
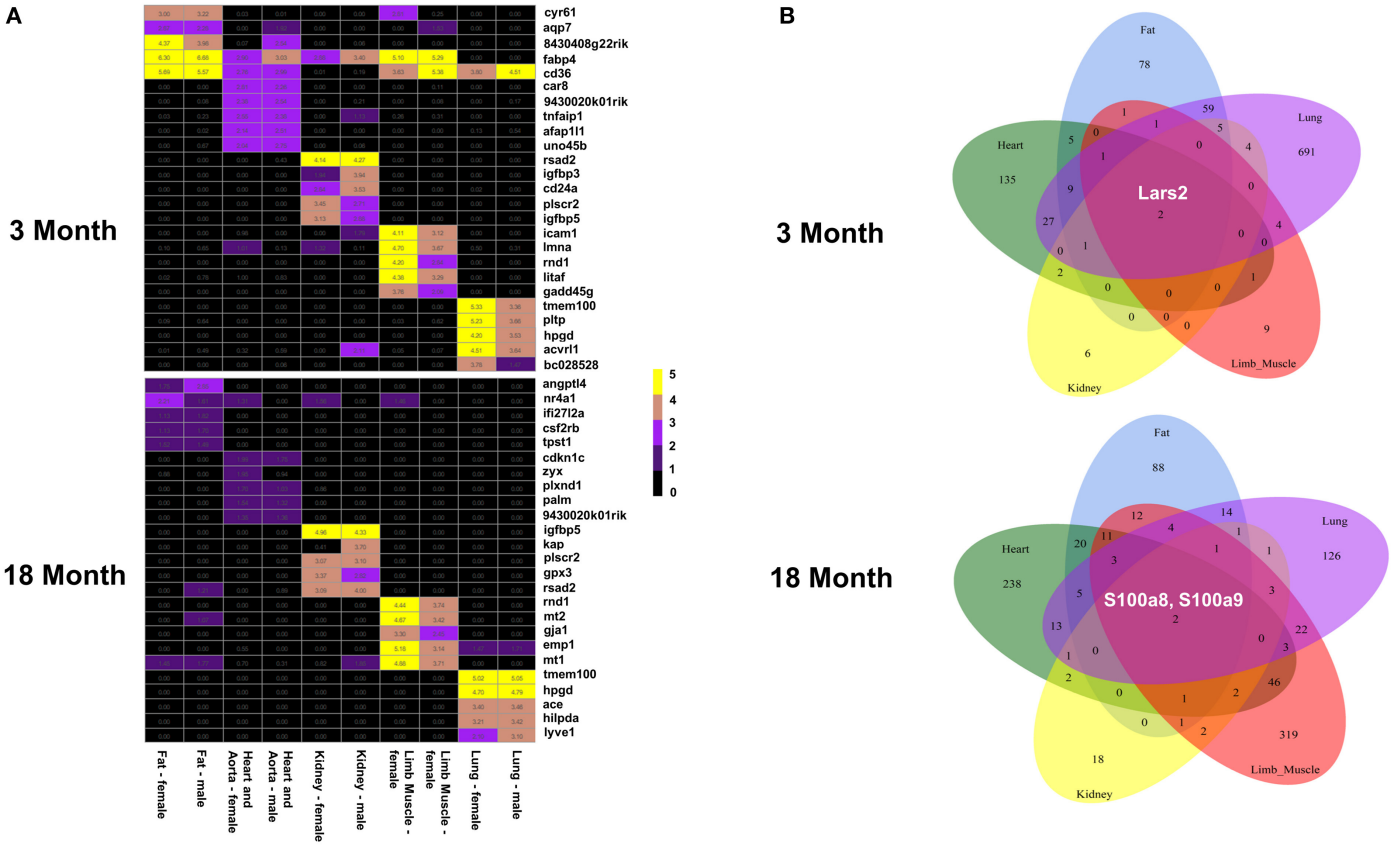
EC gene expression signatures in the five organs. **(A)** Heat map showing the genes with the highest expression values in males and females in fat, heart aorta, kidney, limb and lung at 3 months (top) and 18 months (bottom). **(B)** Venn diagram showing that Lars2 is differentially expressed at 3 months (top) and S100A8 and S100A9 are differentially expressed at 18 months (bottom) in all five organs.

In order to explore if the tissue microenvironment affects the differential expression of ECs, we performed an organ-specific analysis based on age and sex. We found gene expression signatures that distinguish each organ and appear to alter more with age than sex (Figure 3 and Supplementary Figures 2–6, Supplementary Tables 3, 4). Interestingly, we found that the genes Lars2 is differentially expressed in males compared to females in all five organs in the 3-month group. In contrast, S100A8 and S100A9 are upregulated in all organs from males compared to females at 18 months. In addition to various other functions, these genes are involved in regulating immunity and inflammation.

### GO Analysis of DEGS in ECs Based on Age and Sex

**Figure 4:**
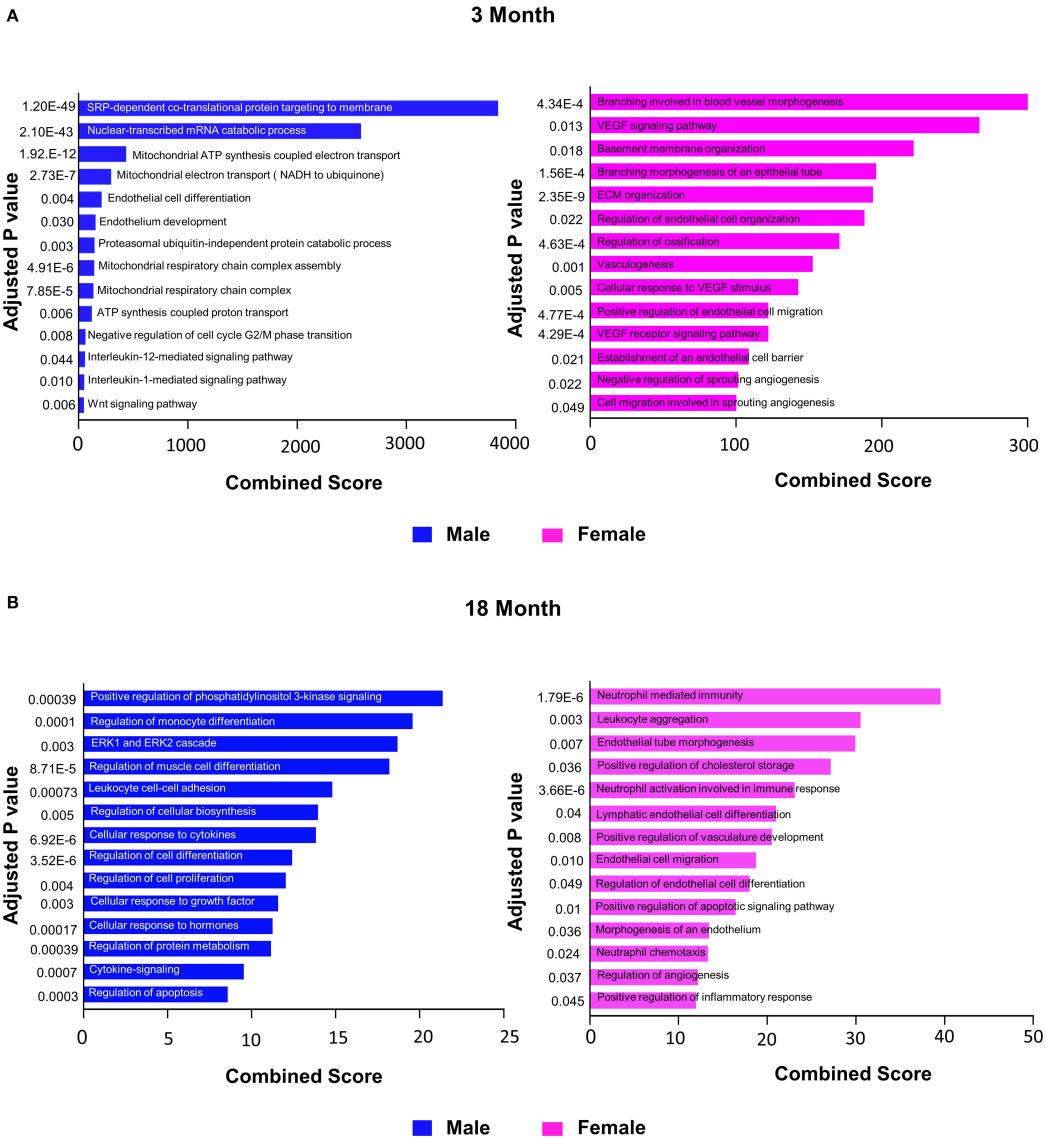
Comparison of biological pathways that are upregulated in ECs from all organs in males and females in the young and old cohort. **(A)** Bar graph showing the top significant biological pathways that are upregulated in males and females at 3 months. **(B)** Bar graph showing significant biological pathways that are upregulated in males and females at 18 months (adjusted *P* < 0.05).

**Figure 5:**
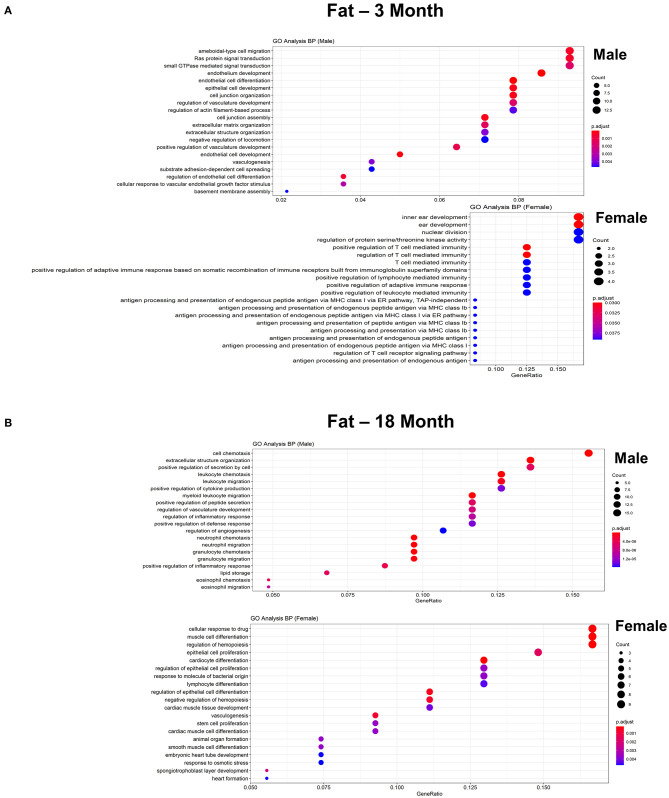
Comparison of the biological pathways that are upregulated in ECs isolated from fat in males and females in the young and old cohort. **(A)** Dot plot showing significant biological pathways that are upregulated in males and females in ECs isolated from fat at 3 months. **(B)** Dot plot showing significant biological pathways that are upregulated in males and females in ECs isolated from fat at 18 months (adjusted *P* < 0.05).

**Figure 6:**
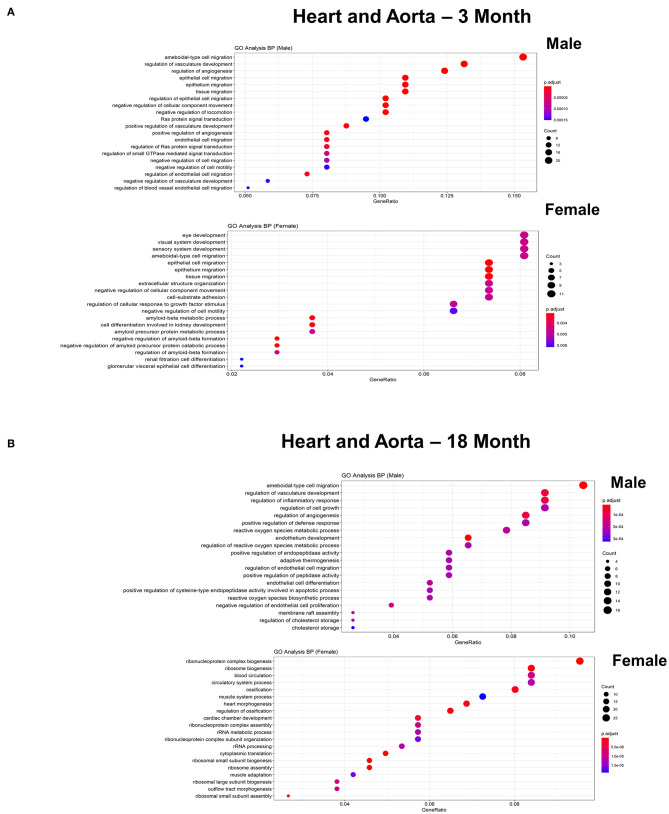
Comparison of the biological pathways that are upregulated in ECs isolated from heart and aorta in males and females in the young and old cohort. **(A)** Dot plot showing significant biological pathways that are upregulated in males and females in ECs isolated from heart and aorta at 3 months. **(B)** Dot plot showing significant biological pathways that are upregulated in males and females in ECs isolated from heart and aorta at 18 months.

**Figure 7:**
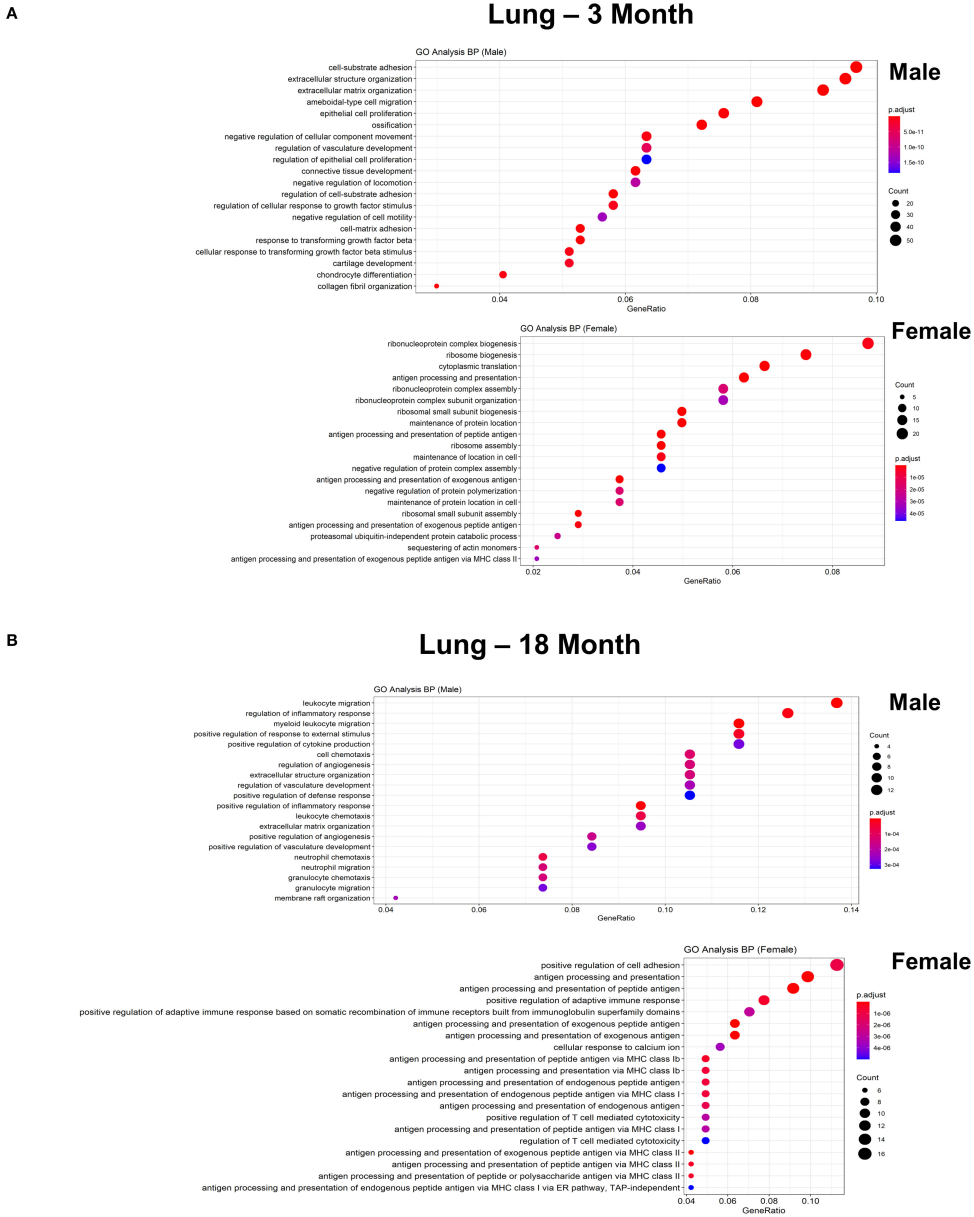
Comparison of biological pathways that are upregulated in ECs isolated from lung in males and females in the young and old cohort. **(A)** Dot plot showing significant biological pathways that are upregulated in males and females in ECs isolated from lung at 3 months. **(B)** Dot plot showing significant pathways that are upregulated in males and females in ECs isolated from lung at 18 months.

**Figure 8:**
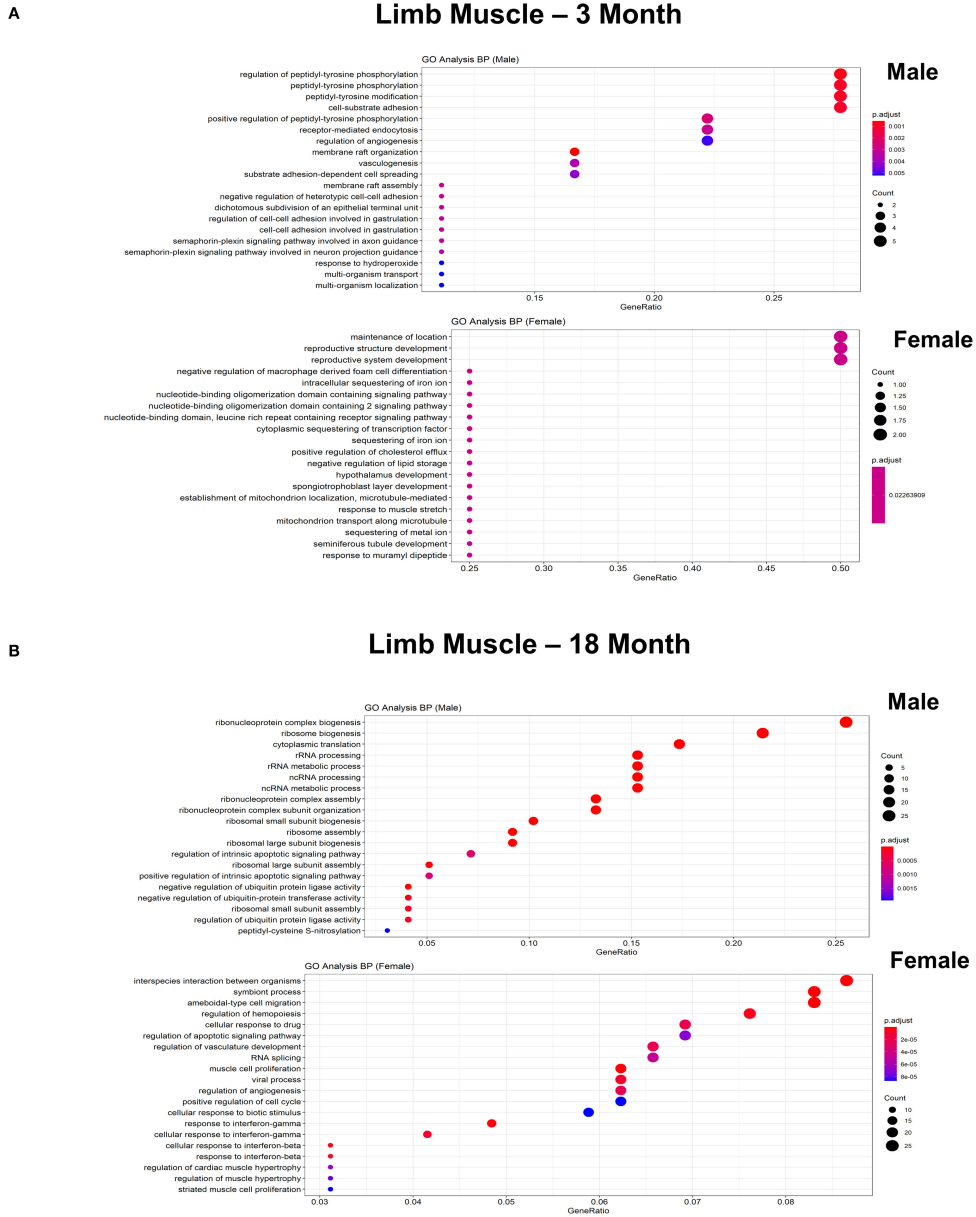
Comparison of biological pathways that are upregulated in ECs isolated from limb muscle in males and females in the young and old cohort. **(A)** Dot plot showing the significant biological pathways that are upregulated in males and females in ECs isolated from limb muscle at 3 months. **(B)** Dot plot showing significant biological pathways that are upregulated in males and females in ECs isolated from limb muscle at 18 months.

**Figure 9:**
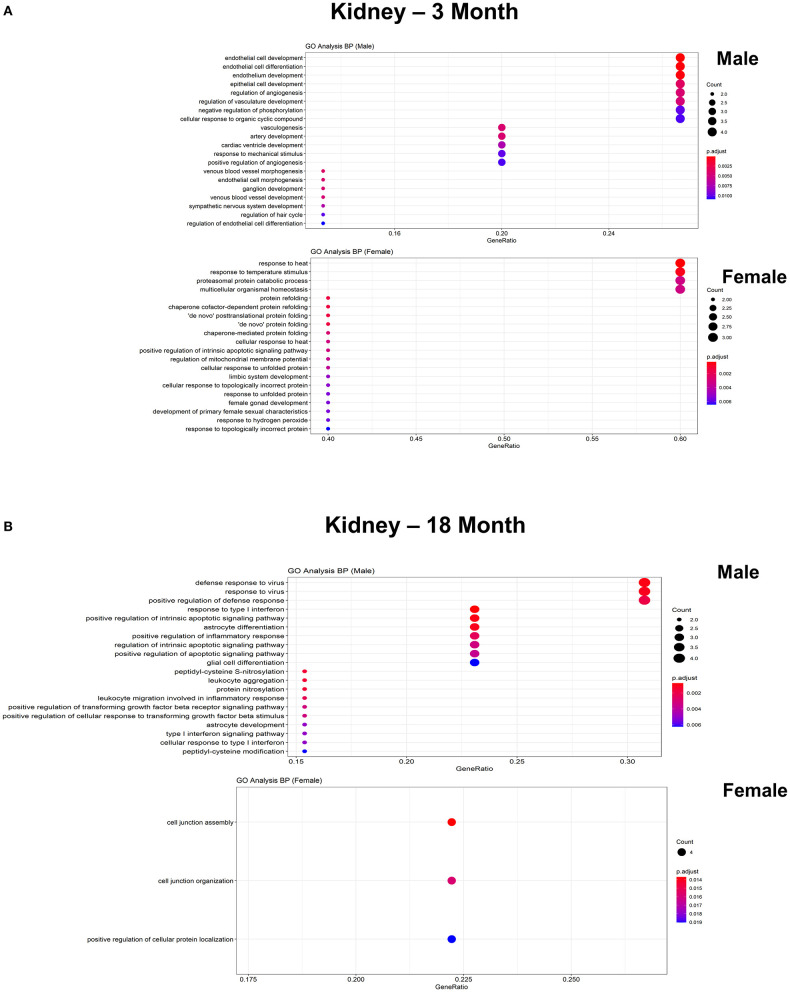
Comparison of biological pathways that are upregulated in ECs isolated from kidney in males and females in the young and old cohort. **(A)** Dot plot showing significant biological pathways that are upregulated in males and females in ECs isolated from kidney at 3 months. **(B)** Dot plot showing significant biological pathways that are upregulated in males and females in ECs isolated from kidney at 18 months.

To further explore differences in functional characteristics of ECs based on age and sex, DEGS were submitted to gene ontology pathway analysis. The overall analysis revealed enrichment in pathways involving protein targeting, catabolism, mitochondrial electron transport, IL 1- and IL 2- signaling, and WNT signaling at 3 months (Figure 4). In contrast, genes involved in angiogenesis and chemotaxis were enriched in females at 3 months. ECs from males and females at 18 months, however, had up-regulation in similar pathways involved in inflammation and apoptosis. When analyzing DEGS stratified by organ (Figures 5–9), we find that genes enriched in pathways regulating inflammation and immunity pathways were upregulated in fat and lung from females. In contrast, these pathways were upregulated in both male and female ECs from fat, in male ECs from heart and aorta, in both male and female ECs from the lung, and in male ECs from the kidney. ECs from limb muscles for both sexes as well as ECs from the heart and aorta were enriched in genes involved in apoptosis.

### Confirmation of Single Cell RNAseq Findings by qPCR and Western Blot

**Figure 10:**
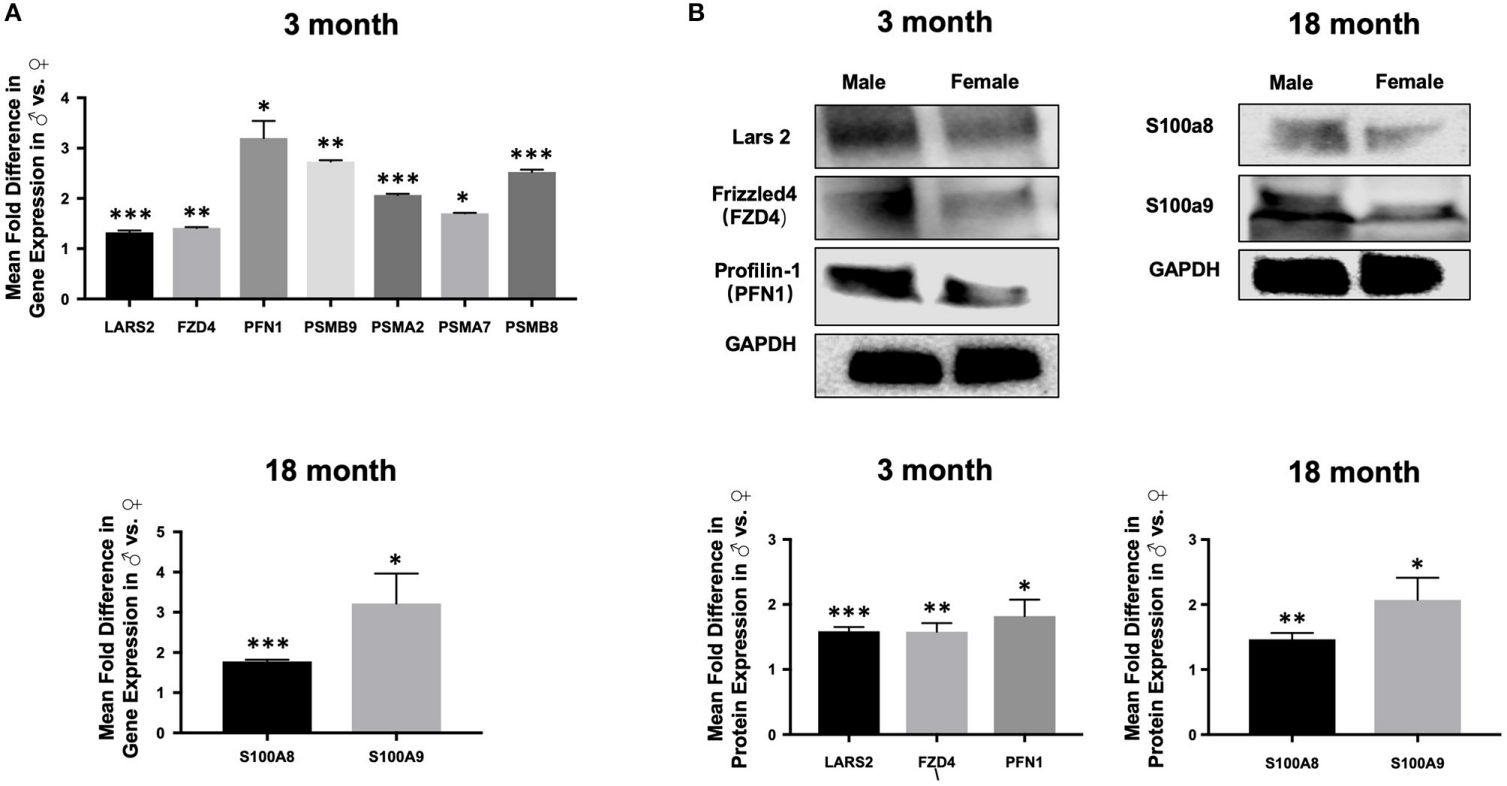
Expression of selected genes and proteins in aortic ECs. **(A)** Bar graphs displaying upregulation of the expression of Lars2 as well as genes involved in the Wnt pathway at 3 months (top) and upregulation of S100A8 and S100A9 at 18-months in males relative to females by quantitative PCR (bottom). **(B)** Western blot analysis confirming the expression of Lars2 as well as genes involved in the Wnt pathway are expressed at the protein level (top) and displayed as relative fold-difference between males and females in a bar graph (bottom). ***P < 0.001; **P < 0.01; and *P < 0.01.

Importantly, findings from RNAseq were verified by qPCR including up-regulation of Lars2 in 3-month old males as well as S100A8 and S100A9 in 18-month old males, respectively (Figure 10). We also confirmed differential expression of genes involved in selected pathways (Figure 10). Further analysis with Western blot showed increased protein expression in Lars2 and selected proteins involved in the Wnt pathway (e.g., FZD4 and PFN1) in 3-month males as well as S100A8 and S100A9 proteins in the 18-month males.

## Discussion

Age and sex are major risk factors for many diseases associated with endothelial dysfunction including obesity (Palmer and Clegg, 2015; Jura and Kozak, 2016), metabolic syndrome (Chella Krishnan et al., 2018), coronary artery disease (Nguyen et al., 2011; Madhavan et al., 2018), diabetes (DECODE Study Group, 2003), hypertension (Gillis and Sullivan, 2016), emphysema (Barnes, 2016), pulmonary artery hypertension (Lakshmanan et al., 2020), sarcopenia (Tay et al., 2015), and chronic kidney disease (Yu et al., 2012). It has been suggested that the female sex chromosome increases survival and lifespan although the exact mechanisms remain unclear (Davis et al., 2019). Biological changes associated with normal chronological aging—including alterations in the immune system, changes in hormone secretion, and defects in the cell repair systems as a result of telomere shortening or cellular mutations—can result in the deterioration of micro- and macrovascular function that can lead to disease development (López-Otín et al., 2013). To evaluate whether age- and sex related differences in EC function are reflected in the EC transcriptome, we performed an unbiased analysis of ECs isolated from fat, heart and aorta, lung, limb muscle, and kidney obtained from the same male and female mice at 3 months (equivalent to the human age of 20–30 years old) and 18 months of age (equivalent to the human age of 50–60 years old).

Our analysis revealed that changes in the EC transcriptome are largely similar between the sexes in each age group. Consistent with previous studies using mouse and human tissues (InanlooRahatloo et al., 2017; Kassam et al., 2019), the relative fold-difference in gene expression of ECs between males and females in the majority of genes is <50% (average fold change <1), a finding that is similar across all organs evaluated. Interestingly, in the entire transcriptome of these organs, Lars2 is the one somatic gene appears to be consistently up-regulated in males compared to females in the younger age group. Lars2 encodes for a mitochondrial leucyl-tRNA synthetase that affect aminoacyl-tRNA ligase activity in mitochondria (‘t Hart et al., 2005; Carminho-Rodrigues et al., 2020). As a part of a unique group of enzymes that catalyzes the ligation of amino acids to their cognate tRNAs, Lars2 as well as other aminoacyl-tRNA synthetases determine the genetic code that is essential for protein synthesis and cell viability. Abnormalities in aminacyl-tRNA synthetases have been implicated in the development of neurological disease, cancer, and auto immune disease (Park et al., 2008). Interestingly, single nucleotide mutations in Lars2, perhaps induced by the accumulation of oxidative stress stimulated by episodes of hyperglycemia and hyperinsulinemia, has been implicated as a novel type 2 diabetes susceptibility gene (Kassam et al., 2019). Mutations in Lars2 have also been associated with sensorineural hearing loss, hydrops, lactic acidosis, sideroblastic anemia, and multisystem failure (Riley et al., 2016; Xia et al., 2018). Although the exact function of Lars2 in ECs is unclear, given its basic function in protein synthesis in mitochondria, further study is warranted to investigate whether the expression levels of Lars2 mediate phenotypic differences between younger males and females.

In contrast to their younger counterparts, across all the organs, the older male mice had upregulation in S100A8 and S100A9 (Vogl et al., 2007), which are Ca^2+^ binding proteins in the S100 family that regulate apoptosis, proliferation, differentiation, migration, energy metabolism, calcium balance, protein phosphorylation, and inflammation. During cellular stress, S100A8 and S100A9 is released as a heterodimer (e.g., calprotectin) into the extracellular space where it binds to TLR4 and initiate a signaling cascade that regulates inflammation, cell proliferation, differentiation, and tumor development in an NF-κB-dependent manner (Turovskaya et al., 2008). Alternatively, calprotectin can interact with receptor for advanced glycation end products (RAGE), which activates NF-κB to induce production of pro-inflammatory cytokines that result in the migration of neutrophils, monocytes, and macrophages (Yen et al., 1997; Sorci et al., 2013). Although predominantly expressed in immune cells, expression of S100A8 and S100A9 is increased in activated endothelial cells under conditions of oxidative stress, hyperglycemia, and pro-inflammatory stimuli (McCormick et al., 2005; Sroussi et al., 2009; Yao and Brownlee, 2010; Furman et al., 2019). Taken together, these findings suggest that age-related changes in the EC tissue microenvironment in males can promote inflammation, which could account for the increased incidence of endothelial dysfunction and its associated diseases among older middle aged males compared to their female counterparts.

Consistent with these findings, we found that the differentially expressed genes in older mice were enriched in pathways related to inflammation. Aging has long been associated with the development of inflammation (Donato et al., 2015). Previous studies have demonstrated that aging endothelial cells acquire a senescent phenotype characterized by increased secretion of pro-inflammatory cytokines and chemokines into the micro-environment (Hoffmann et al., 2001; Uraoka et al., 2011). Previous studies have shown that senescent endothelial cells do not migrate, proliferate, or sprout; they have limited capacity to form new vessels and have reduced numbers of endothelial progenitor cells; and they do not respond appropriately to hypoxia (e.g., reduced expression of HIF-1 alpha and angiogenic factors) (Lin et al., 2015; Rudnicki et al., 2018). These senescent cells contribute to many non-communicable age-related chronic diseases including insulin resistance, CVD, pulmonary arterial hypertension, chronic obstructive pulmonary disorder, emphysema, Alzheimer's and Parkinson's diseases, macular degeneration, osteoarthritis, and cancer (Lin et al., 2015). Although the exact reasons why these cells develop this senescent phenotype is unclear, studies suggest that both endogenous factors related to biological aging (e.g., oxidative stress, telomere shortening, and DNA damage) and environmental factors (e.g., diet, stress, and chronic infection) may contribute (Uraoka et al., 2011).

Unlike their older counterparts, younger female mice had activation of pathways associated with angiogenesis including activation of genes involved in blood vessel morphogenesis, VEGF signaling, and endothelial cell migration and organization. This finding is consistent with previous studies that have shown that young female mice produce higher levels of proangiogenic factors and vascularity in response to stress than male mice (Xu et al., 2019). Angiogenesis is an important adaptive response to physiological stress and an endogenous repair mechanism after injury that can be impaired with aging. In contrast to young female mice, young male mice showed increased expression of genes involved in the Wnt signaling pathway, which has been shown to be an important regulator of lifespan, especially in the earlier stages of life (MacDonald et al., 2009; Franco et al., 2016). In endothelial cells, Wnt ligands have been shown to regulate vascular remodeling through their regulation of endothelial cell survival and proliferation (MacDonald et al., 2009). Although further study is needed, these findings suggest that vascular morphogenesis in males and females are regulated by diverse pathways.

In summary, our unbiased, integrated analysis of the gene transcriptome has revealed that the EC transcriptome is largely similar in male and female mice. Older mice, especially males, have increased expression of genes involved in immunity and inflammation, which could contribute to the increased prevalence of age-related non-communicable diseases associated with endothelial dysfunction in older men. Future studies are needed to further elucidate the role of DEGS identified in this study in the development of disease.

### Limitations

The major limitation of this study is that not all of the organs were collected from both males and females in both age groups. The five organs that we analyzed, however, represent major tissues with important physiological function for health. Another limitation is that single cell sequencing was performed using different techniques for the young (e.g., plate-seq) and old group (e.g., dropseq). In the *Tabula Muris and Tabula Muris Senis* project, gene expression data from 20 organs were performed using these two sequencing methods and compared. The study showed close agreement between the genes, defining each organ-specific cells cluster for both methods. Moreover, gene expression analysis showed several hundred genes were differentially expressed to a similar degrees across organs using both methods. To address the differences in sequencing methods, in our study, we perform DEG and pathway analysis separately for each age group. Within each age group, we calculated the relatively gene expression only for males and females. Any comparisons between age groups was performed only on the output of the differential analysis. Importantly, we performed qPCR on selected genes to confirm results from the RNAseq analysis.

## Data Availability Statement

Publicly available datasets were analyzed in this study. This data can be found at: https://figshare.com/projects/Tabula_Muris_Transcriptomic_characterization_of_20_organs_and_tissues_from_Mus_musculus_at_single_cell_resolution/27733.

## Ethics Statement

This animal study was reviewed and approved by Stanford APLAC.

## Author Contributions

PN: conceptualization and supervision. WS, SV, and XH: methodology. WS: data curation. NS: qPCR and Western blot. GL: writing and reviewing. XH and PN: writing review and editing. All authors contributed to the article and approved the submitted version.

## Conflict of Interest

The authors declare that the research was conducted in the absence of any commercial or financial relationships that could be construed as a potential conflict of interest.

## Supplementary Material

Supplementary Figure 1 Schematic of study. **(A)** Endothelial cells (ECs) defined by expression of PECAM and Cdh5 were identified from the Tabula Muris dataset and analyzed by organ specific expression, by age group, and by sex. **(B)** Five organs from 6 males (4 at 3 months and 2 at 18 months) and 7 females (3 at 3 months and 4 at 18 months) were analyzed. Data for 3 and 18 month were generated from single cell plateseq and dropseq data from the Tabula Muris Project, respectively.

Supplementary Figure 2 Age and sex differences in gene expression in ECs from fat. **(A)** *T-SNE* visualization of endothelial cells from fat. **(B)** Violin plots showing the top genes, defined by their fold change, which were differentially expressed in fat in males vs. females and in young vs. old.

Supplementary Figure 3 Age and sex differences in gene expression in ECS from the heart and aorta. **(A)** *T-SNE* visualization of endothelial cells from heart and aorta. **(B)** Violin plots showing the top genes that were differentially expressed in heart and aorta in males vs. females and in young vs. old.

Supplementary Figure 4 Age and sex differences in gene expression in ECS from the lung. **(A)** *T-SNE* visualization of endothelial cells from lung. **(B)** Violin plots showing the top genes that were differentially expressed in lung in males vs. females and in young vs. old.

Supplementary Figure 5 Age and sex differences in gene expression in ECS from the limb muscle. **(A)** *T-SNE* visualization of endothelial cells from limb muscle. **(B)** Violin plots showing the top genes that were differentially expressed in limb muscle in males vs. females and in young vs. old.

Supplementary Figure 6 Age and sex differences in gene expression in ECS from kidney. **(A)** *T-SNE* visualization of endothelial cells from kidney. **(B)** Violin plots showing the top genes that were differentially expressed in kidney in males vs. females and in young vs. old.

Supplementary Table 1 Genes differentially expressed in both 3 month and 18 month groups.

Supplementary Table 2 Organ-specific gene signatures.

Supplementary Table 3 Significant difference in gene expression in each organ between male and female in 3 Month.

Supplementary Table 4 Significant difference in gene expression in each organ between male and female in 18 Month.
