# Supplementary figures and images for: Single-Cell Transcriptional Profiling Reveals Sex and Age Diversity of Gene Expression in Mouse Endothelial Cells
Source: Front Genet. 2021 Feb 17;12:590377. doi: 10.3389/fgene.2021.590377 (PMC7929607; doi:10.3389/fgene.2021.590377)

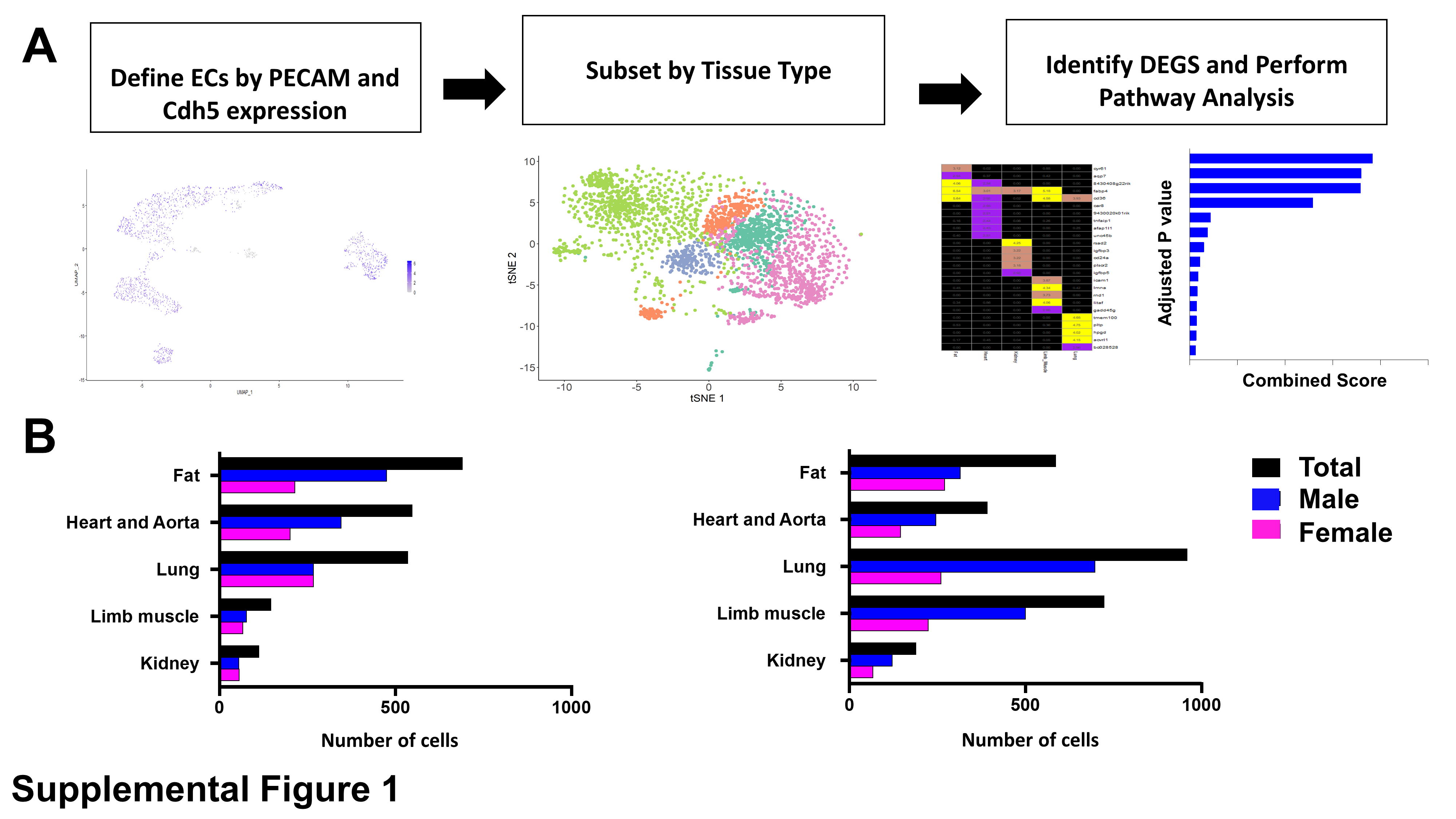

Supplement: Supplementary Figure 1 — Schematic of study. (A) Endothelial cells (ECs) defined by expression of PECAM and Cdh5 were identified from the Tabula Muris dataset and analyzed by organ specific expression, by age group, and by sex. (B) Five organs from 6 males (4 at 3 months and 2 at 18 months) and 7 females (3 at 3 months and 4 at 18 months) were analyzed. Data for 3 and 18 month were generated from single cell plateseq and dropseq data from the Tabula Muris Project, respectively. [file Image_1.TIF]

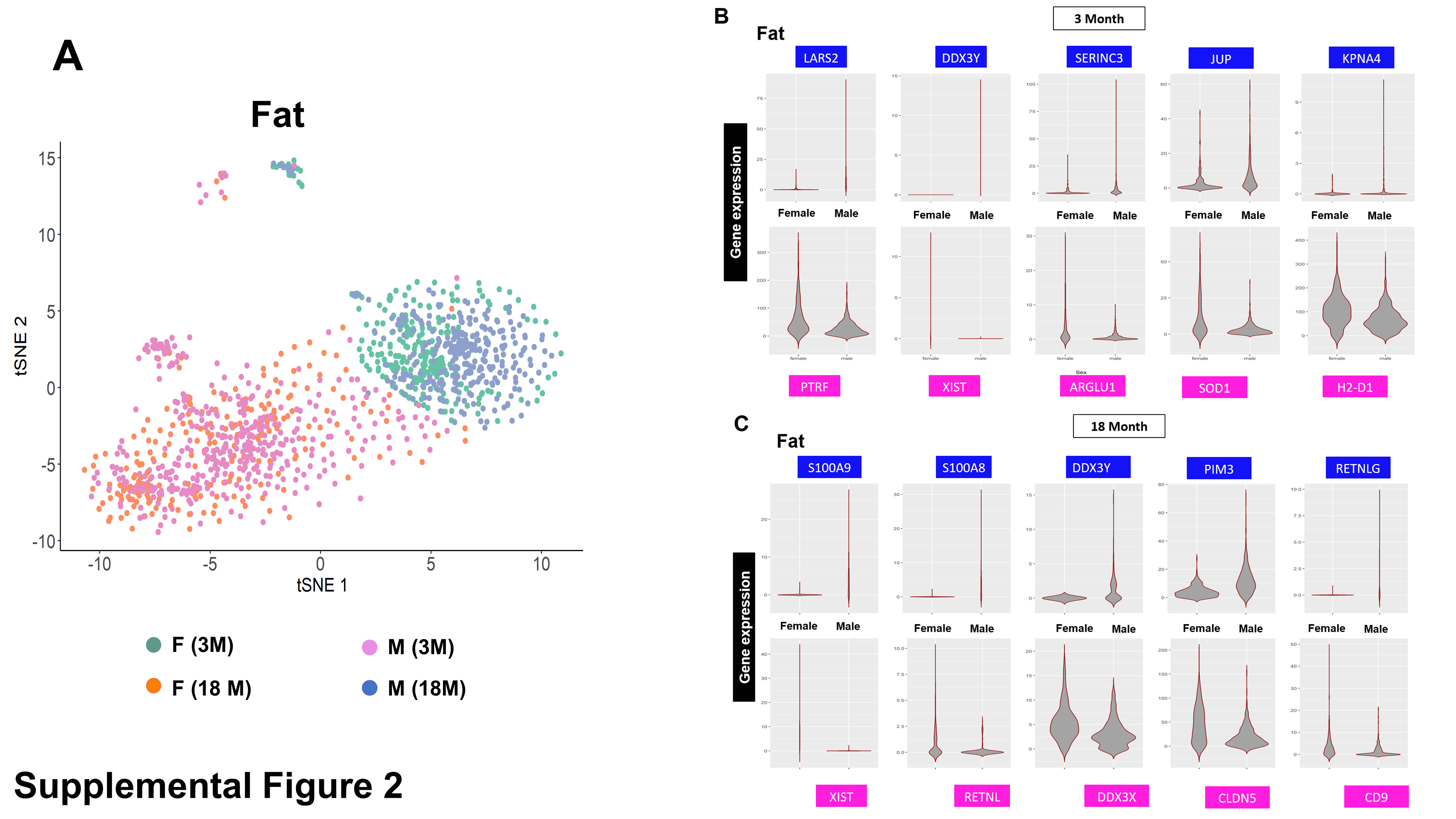

Supplement: Supplementary Figure 2 — Age and sex differences in gene expression in ECs from fat. (A) T-SNE visualization of endothelial cells from fat. (B) Violin plots showing the top genes, defined by their fold change, which were differentially expressed in fat in males vs. females and in young vs. old. [file Image_2.TIF]

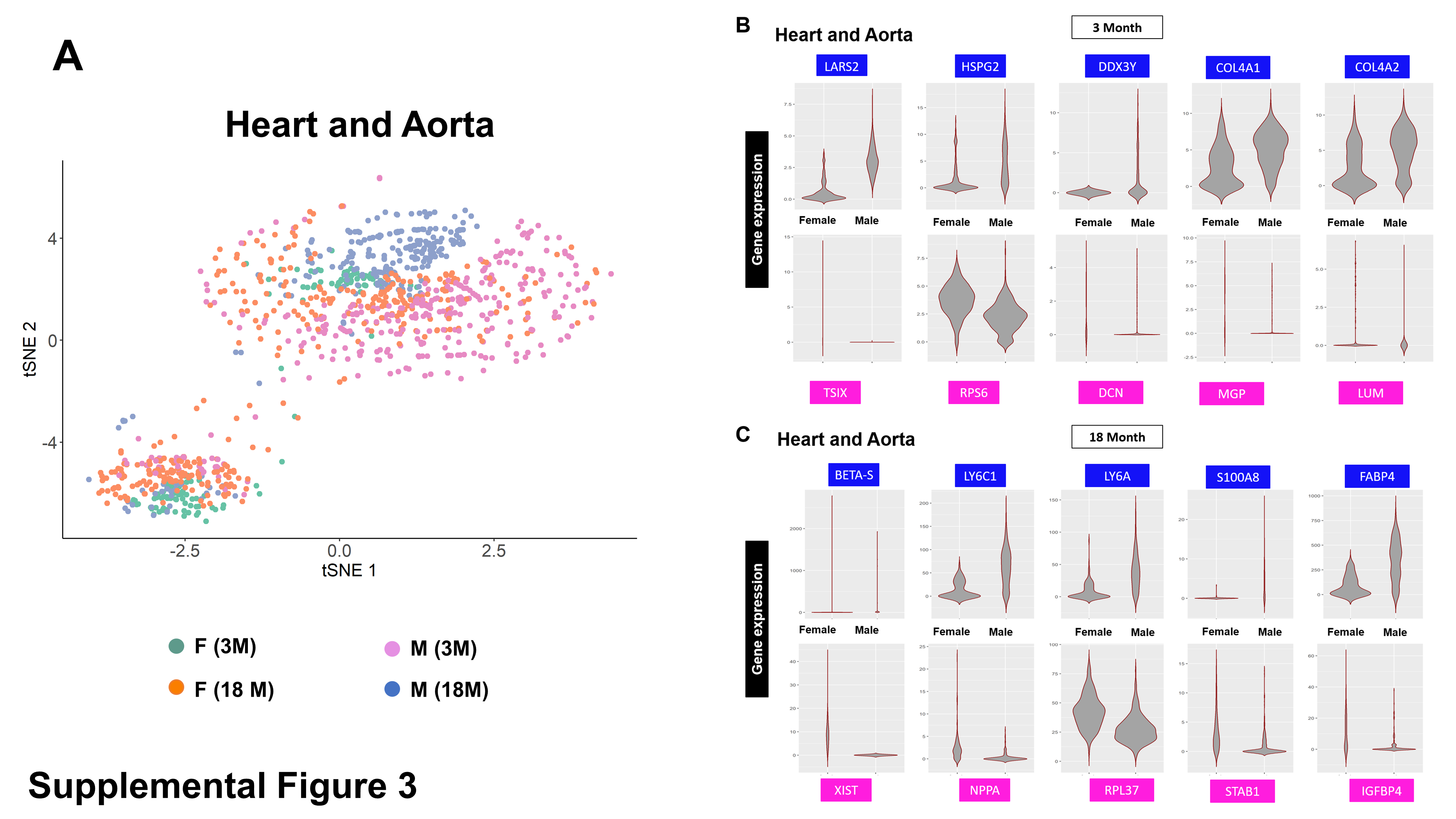

Supplement: Supplementary Figure 3 — Age and sex differences in gene expression in ECS from the heart and aorta. (A) T-SNE visualization of endothelial cells from heart and aorta. (B) Violin plots showing the top genes that were differentially expressed in heart and aorta in males vs. females and in young vs. old. [file Image_3.TIF]

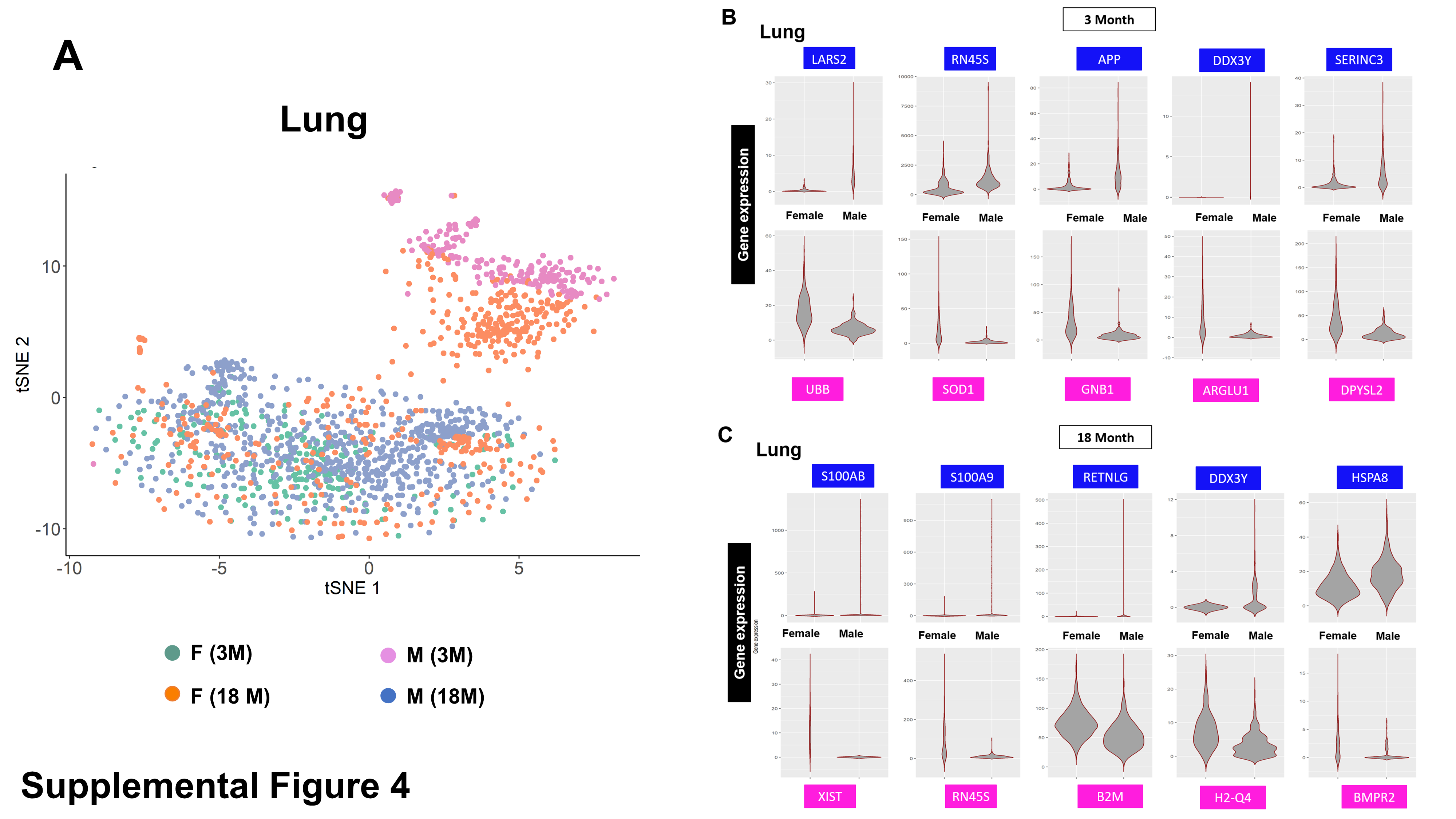

Supplement: Supplementary Figure 4 — Age and sex differences in gene expression in ECS from the lung. (A) T-SNE visualization of endothelial cells from lung. (B) Violin plots showing the top genes that were differentially expressed in lung in males vs. females and in young vs. old. [file Image_4.TIF]

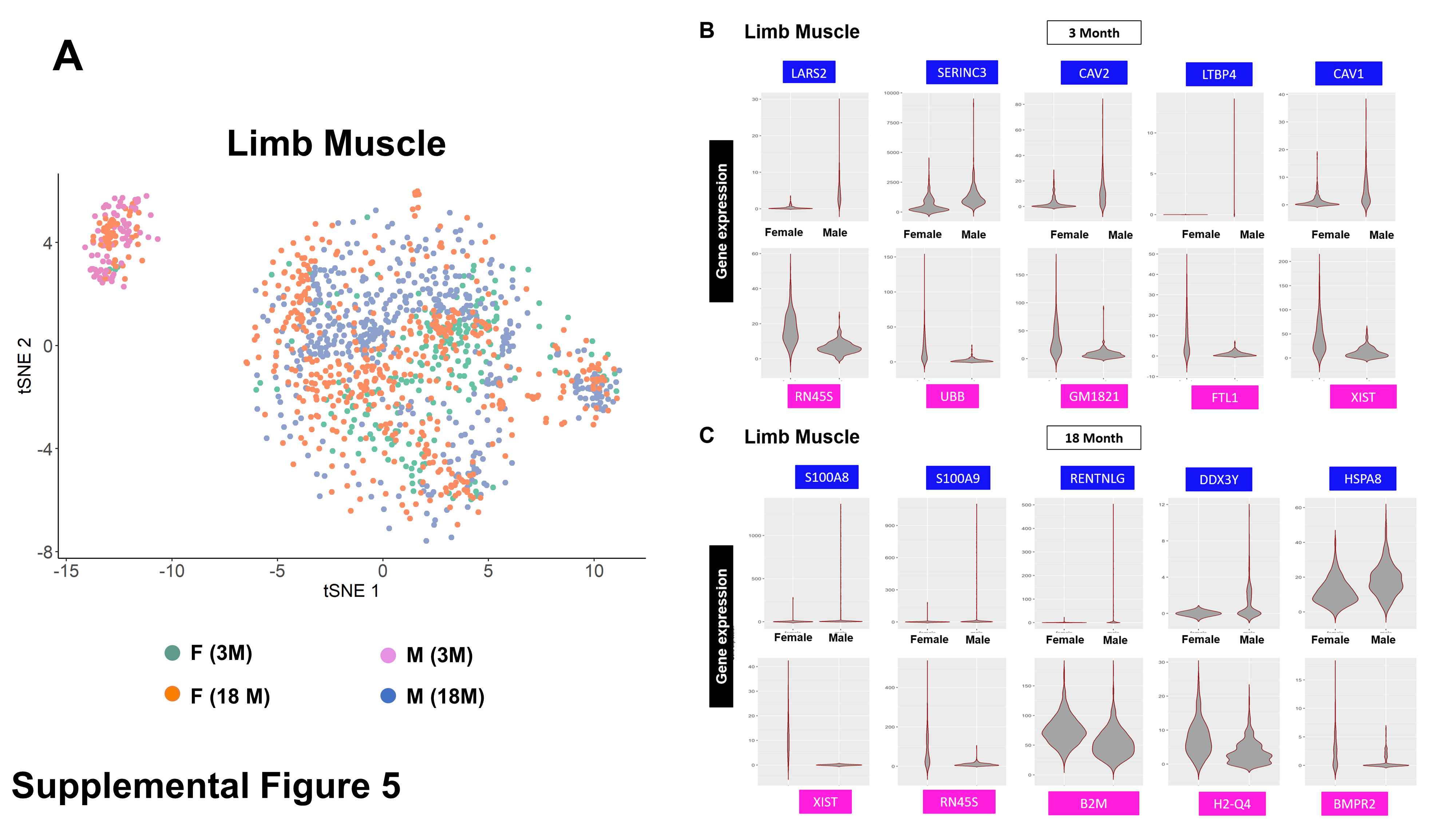

Supplement: Supplementary Figure 5 — Age and sex differences in gene expression in ECS from the limb muscle. (A) T-SNE visualization of endothelial cells from limb muscle. (B) Violin plots showing the top genes that were differentially expressed in limb muscle in males vs. females and in young vs. old. [file Image_5.TIF]

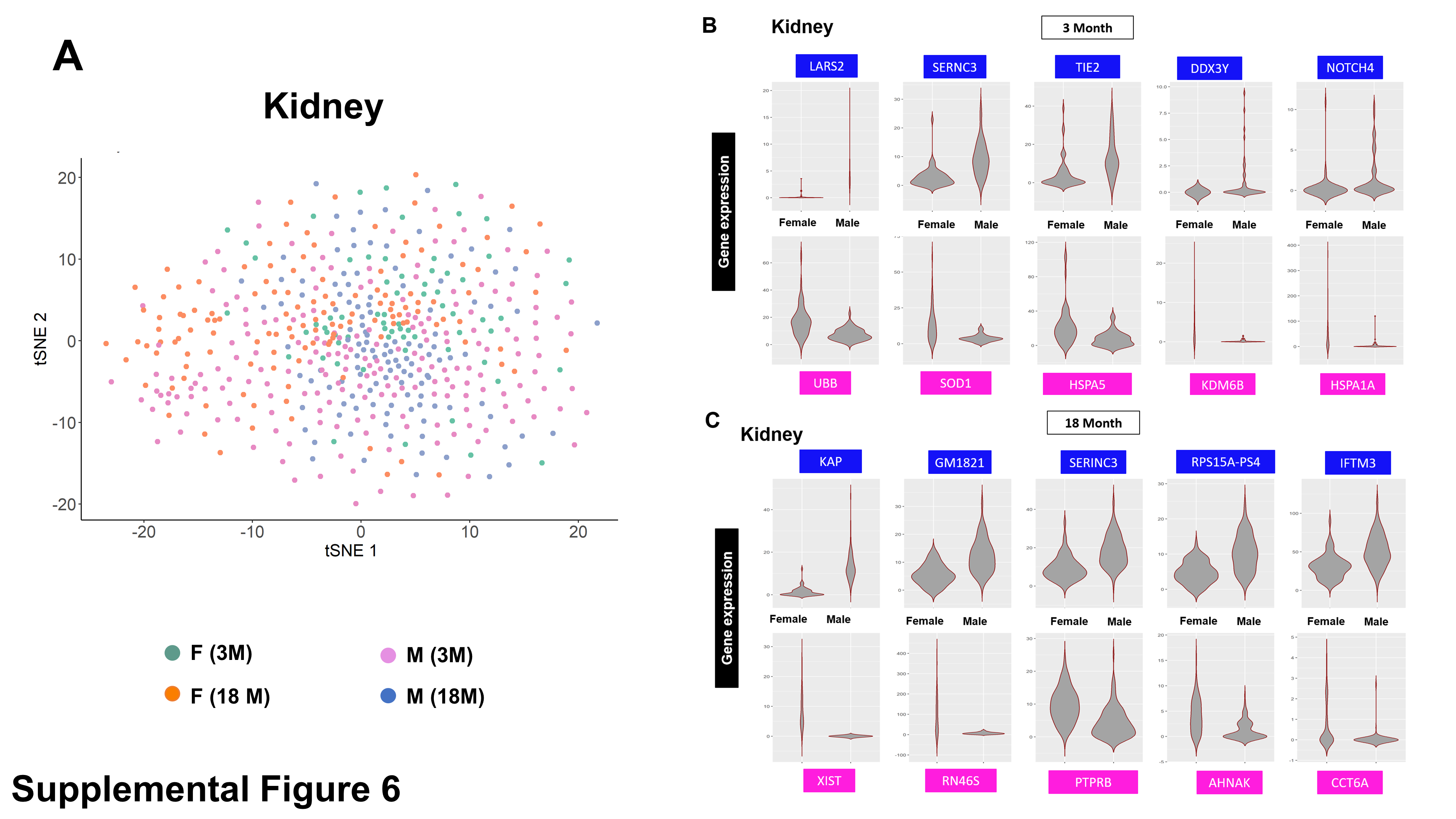

Supplement: Supplementary Figure 6 — Age and sex differences in gene expression in ECS from kidney. (A) T-SNE visualization of endothelial cells from kidney. (B) Violin plots showing the top genes that were differentially expressed in kidney in males vs. females and in young vs. old. [file Image_6.TIF]
